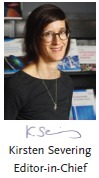# *Advanced Science* – On the Route to Open Research

**DOI:** 10.1002/advs.201903436

**Published:** 2020-01-08

**Authors:** Kirsten Severing

In conversations with our readers and authors, we have been noticing an increasing demand for *Open Research* for several years. This demand is further intensified by requests of funding organizations as well as politics. *Open Research* is a very broad term that covers various aspects of science becoming more open, accessible, efficient, democratic, and transparent, as defined by the European Commission. Certainly, publishers and journals play an essential role in this process. We do not only consider *Advanced Science* at the forefront of this development, but we are also committed to further boost this process.

Manuscripts published in an *Open Access*‐journal such as *Advanced Science* are accessible to everyone anywhere at any time. They are easy to find on our journal‐website as well as in all important indexing services (e.g., PubMedCentral, Web of Knowledge etc.). Publishing under a CC‐BY license accelerates and simplifies communication. Furthermore, we accept submission of papers published on preprint‐servers in order to improve rapid research availability and transparency. The visibility of your results can be further enhanced by ordering a Video Abstract (https://onlinelibrary.wiley.com/page/journal/21983844/homepage/video-abstract-gallery.html) or promotion in various social networks.

The exceptional quality of our publications is guaranteed by a fast, rigorous and strict peer‐review process. Starting in 2020, our referees will have the option to receive ORCID‐reviewer recognition. This is realized in a very convenient and automated way and represents an additional step towards *Open Research*. As a result, we are confident that our ongoing ambition to facilitate interdisciplinary collaborative science and efficient impactful research will continue to be prosperous.

**Figure 1:**
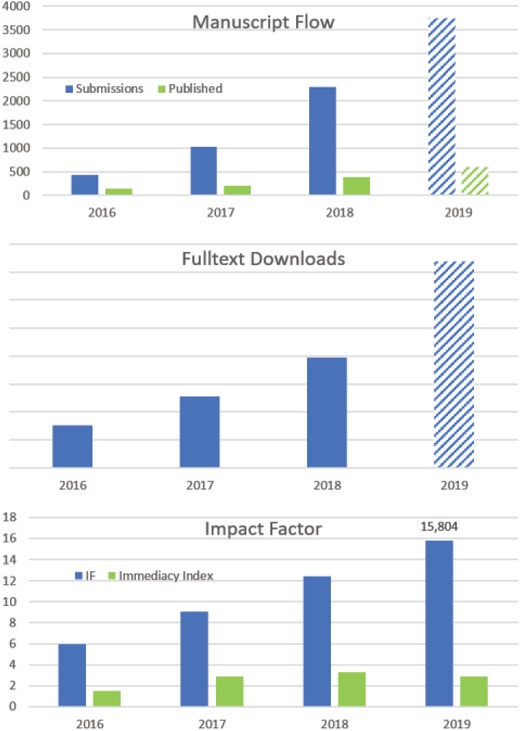
Key performance indicators for *Advanced Science* 2016 to 2019. Data for 2019 are projections based on numbers until mid November.

The key performance indicators for *Advanced Science* illustrate that our way towards *Open Research* is going in the right direction. Submissions increased by more than 60% and the number of published papers increased by more than 35% compared to the previous year (both values at the time of writing 2019). The impact factor raised to a record value of currently 15.804, which represents an increase of 27% compared to 2018. Overall the number of downloaded articles nearly doubled within the last 12 months. **Figure** **1** shows this continuing and lasting trend in a striking way.

On the occasion of our 5^th^ anniversary in 2019, we invited our executive advisory board members to contribute articles for a celebratory series to be published in a continuously expanding virtual issue (https://onlinelibrary.wiley.com/doi/toc/10.1002/(ISSN)2198-3844.AnniversaryVirtualIssue). These articles showcase outstanding achievements of leading international researchers in the field of materials science, physics and chemistry, medical and life sciences, as well as engineering. In case you are interested in getting to know our executive advisory board members on a more personal level, I would like to recommend our interview‐based highlights on our website Advanced Science News (https://www.advancedsciencenews.com/tag/advanced-science-5th-anniversary/).


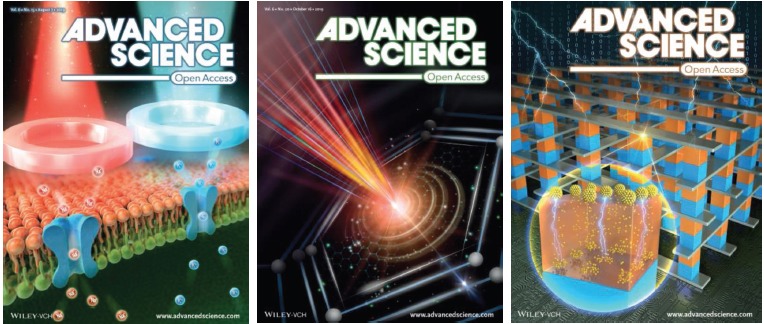


**Table** **1** shows the papers with the strongest influence on the coming impact factor in 2020. These publications are mainly from energy related topics, like batteries, supercapacitors, and water splitting. **Table** **2**, on the other hand represents those *Advanced Science* articles published in 2019 that received the highest Altmetric score. This number is an indication of the attention that a publication gained, with red color indicating mainstream media coverage on news platforms, yellow indicating blogs, and blue representing Twitter activities. The table nicely reflects the broad topical scope of our journal with research on 3D printed hearts, or bacterial resistance and new insights into Alzheimer's disease, to more material science‐oriented topics on energy storage or the worlds' thinnest gold.

**Table 1:** Highest cited manuscripts in 2019

Manuscript Title	Corresponding Author(s), Affiliation(s)	Publication Date	Cited in 2019	Total Citations
https://onlinelibrary.wiley.com/doi/full/10.1002/advs.201600539	Jinping Liu, Yuanyuan Li et al., Wuhan University of Technology and Huazhong University of Science & Technology, China	July 2017	179	314
https://onlinelibrary.wiley.com/doi/10.1002/advs.201600243	Xiaobo Ji at al., Central South University, China	January 2017	102	187
https://onlinelibrary.wiley.com/doi/10.1002/advs.201600190	Pooi See Lee at al., Nanyang Technology University, Singapore	February 2017	111	176
https://onlinelibrary.wiley.com/doi/full/10.1002/advs.201700464	Ji‐Jun Zou et al., Tianjin University, China	February 2018	107	144
https://onlinelibrary.wiley.com/doi/full/10.1002/advs.201600337	Ming‐Yong Han et al., Agency for Science, Technology and Research, Singapore	May 2017	97	174
https://onlinelibrary.wiley.com/doi/10.1002/advs.201700691	Bao Yu Xia et al., Huazhong University of Science & Technology, China	April 2018	96	136
https://onlinelibrary.wiley.com/doi/10.1002/advs.201700270	Quan‐Hong Yang, Wie Lv et al., Tianjin University and Tsinghua University, China	January 2018	80	110
https://onlinelibrary.wiley.com/doi/10.1002/advs.201600408	Zhen Zhou et al., Nankai University, China	August 2017	78	125
https://onlinelibrary.wiley.com/doi/full/10.1002/advs.201600445	Qiang Zhang, Yu‐Guo Guo et al., Tsinghua University, University of Chinese Academy of Science, Beijing, China	March 2017	83	184
https://onlinelibrary.wiley.com/doi/full/10.1002/advs.201700592	Huan Pang et al., Yangzhou University, China	March 2018	78	100

**Table 2:**
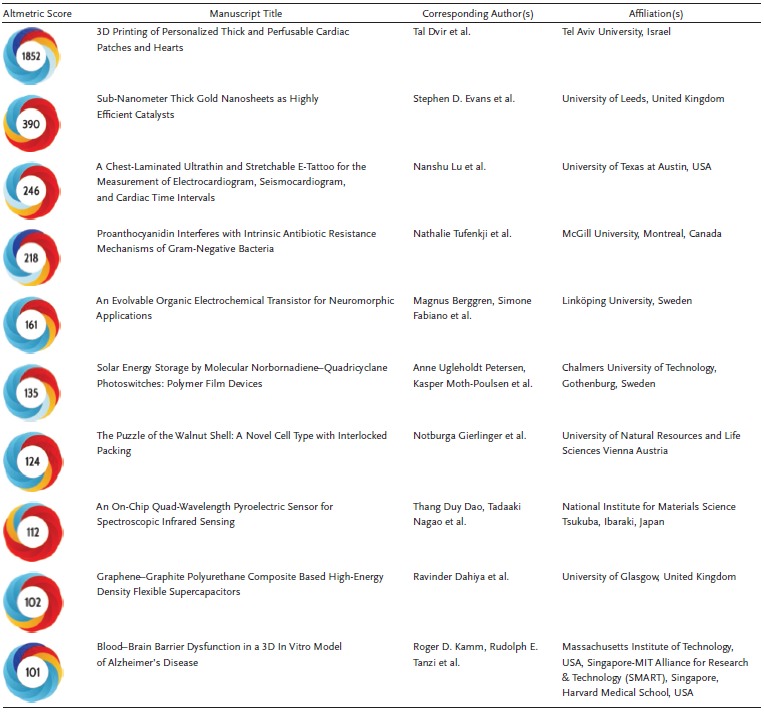
Manuscripts with highest media attention (Altmetric Score)

With all this growth, more dedicated people are needed to run the journal. I am happy to announce that we have 2 new Deputy Editors (supporting Anne Pfisterer, Prisca Henheik, and me): Ulf Scheffler was promoted to a Deputy Editor position in *Advanced Science* already in summer 2019. Bo Weng from our Beijing office joins the team as a new Deputy Editor for the new year. This growing team is happy to receive feedback and suggestions, and of course looking forward to an interesting and successful new year full of exciting research submitted to *Advanced Science*.

Within only a few years, *Advanced Science* has evolved into an impactful, well‐known, and eminently respectable journal that publishes the best international research from all areas of natural science. This would certainly not have been possible without the tremendous support by our readers, executive board members, reviewers, and authors. We would like to thank you and we are looking forward to a great future.

On behalf of the whole editorial team,